# Complete absence of GLUT1 does not impair human terminal erythroid differentiation^∗^

**DOI:** 10.1182/bloodadvances.2024012743

**Published:** 2024-06-26

**Authors:** Catarina Martins Freire, Nadine R. King, Monika Dzieciatkowska, Daniel Stephenson, Pedro L. Moura, Johannes G. G. Dobbe, Geert J. Streekstra, Angelo D'Alessandro, Ashley M. Toye, Timothy J. Satchwell

**Affiliations:** 1 School of Biochemistry, University of Bristol, Bristol, United Kingdom; 2 Department of Biochemistry and Molecular Genetics, University of Colorado Anschutz Medical Campus, Aurora, CO; 3 Department of Medicine, Center for Haematology and Regenerative Medicine, Karolinska Institutet, Huddinge, Sweden; 4 Biomedical Engineering and Physics, University of Amsterdam, Amsterdam UMC location, Amsterdam, The Netherlands

## Abstract

The glucose transporter 1 (GLUT1) is 1 of the most abundant proteins within the erythrocyte membrane and is required for glucose and dehydroascorbic acid (vitamin C precursor) transport. It is widely recognized as a key protein for red cell structure, function, and metabolism. Previous reports highlighted the importance of GLUT1 activity within these uniquely glycolysis-dependent cells, in particular for increasing antioxidant capacity needed to avoid irreversible damage from oxidative stress in humans. However, studies of glucose transporter roles in erythroid cells are complicated by species-specific differences between humans and mice. Here, using CRISPR–mediated gene editing of immortalized erythroblasts and adult CD34^+^ hematopoietic progenitor cells, we generate committed human erythroid cells completely deficient in expression of GLUT1. We show that absence of GLUT1 does not impede human erythroblast proliferation, differentiation, or enucleation. This work demonstrates, to our knowledge, for the first time, generation of enucleated human reticulocytes lacking GLUT1. The GLUT1–deficient reticulocytes possess no tangible alterations to membrane composition or deformability in reticulocytes. Metabolomic analyses of GLUT1–deficient reticulocytes reveal hallmarks of reduced glucose import, downregulated metabolic processes and upregulated AMP-activated protein kinase signaling, alongside alterations in antioxidant metabolism, resulting in increased osmotic fragility and metabolic shifts indicative of higher oxidant stress. Despite detectable metabolic changes in GLUT1–deficient reticulocytes, the absence of developmental phenotype, detectable proteomic compensation, or impaired deformability comprehensively alters our understanding of the role of GLUT1 in red blood cell structure, function, and metabolism. It also provides cell biological evidence supporting clinical consensus that reduced GLUT1 expression does not cause anemia in GLUT1–deficiency syndrome.

## Key Points

• GLUT1 knockout does not impair erythroid differentiation and minimally affects reticulocyte membrane composition.
• Metabolic adaptation facilitates reticulocyte tolerance of GLUT1 absence.

## Visual Abstract

**Figure:**
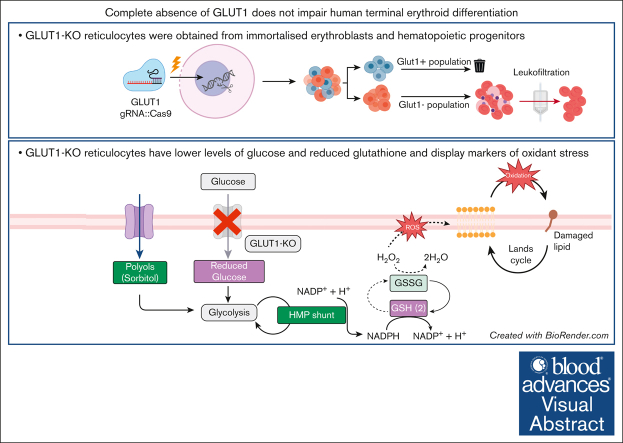

## Introduction

Glucose is an essential source of energy, sustaining life across multiple organisms. Glucose transport in vertebrates is assured by the SLC2 (GLUT) family, with GLUT1, encoded by the *SLC2A1* gene, as the first characterized glucose transporter.^1^ Of all the cell lineages within the human body, human erythrocytes express the highest levels of the GLUT1 transporter, with ∼200 000 copies per cell, accounting for 10% of the total membrane protein mass of the erythrocyte.^2^

Within the red blood cell (RBC) membrane, GLUT1 is located in the junctional multiprotein complex, in which it associates with the underlying spectrin cytoskeleton via cytoskeletal adapter proteins such as dematin and adducin.^3^^,^^4^ GLUT1 also interacts with stomatin,^5^ a membrane-associated protein that binds to cholesterol and is involved in membrane scaffolding,^6^ and GLUT1 1 is proposed to associate with band 3 (anion exchanger 1) via its C-terminal domain.

GLUT1 transports glucose into erythroid cells, providing a source of energy for adenosine triphosphate (ATP) generation either through the Krebs cycle during erythropoiesis or via maintaining glycolysis in the mature RBCs after the loss of mitochondria that occurs during terminal differentiation.^7^ Characteristic features of RBCs such as their extensive capacity for deformation during capillary transit, influenced by ATP-dependent phosphorylation, calcium, and cell volume homeostasis, are intrinsically linked to the metabolic status of the erythrocyte, and therefore governed by the availability of glucose.^8^^,^^9^ Somewhat paradoxically, although RBCs are exclusively reliant upon anaerobic glycolysis to meet their energetic requirements, glucose transport decreases during erythropoiesis (despite increased expression of GLUT1), because of a shift to the transport of the vitamin C precursor dehydroascorbic acid (DHA) favored in humans and other primates that lack the ability to synthesize the potent antioxidant ascorbic acid (vitamin C).^10^ The alteration in GLUT1 transport substrate selectivity is driven by the association of stomatin.^10^

The complete absence of GLUT1 in humans has not been reported and is presumed to be embryonically lethal. GLUT1 deficiency syndrome (G1DS) is a neurodevelopmental disorder that results from haploinsuffiency for GLUT1 due to specific mutations in the *SLC2A1* gene. This leads to impaired glucose transport into the brain and RBCs,^11^ manifesting as a range of neurological symptoms, including epilepsy, developmental delay, and movement disorders.^12^, ^13^, ^14^ The neurological disease can be modeled in mice by introducing heterozygous GLUT1 mutations,^15^ but because GLUT1 is only expressed in erythrocytes during the murine perinatal period and is rapidly replaced by GLUT4 in adult mouse erythrocytes, the impact of haploinsufficiency of GLUT1 on RBCs cannot be fully modeled in mice. Interestingly, despite the perceived pivotal role of GLUT1 in RBC function, patients with G1DS typically do not exhibit any hematological phenotypes arising from the deficiency of this protein.^16^ The exact reason for this “lack of RBC phenotype” remains unclear and represents an intriguing area of investigation.

In this study we exploit CRISPR–mediated gene editing of both immortalized erythroblasts and adult hematopoietic stem cells, generating novel GLUT1 knockout (KO) erythroid cells to establish the impact of total loss of GLUT1 on RBC formation, membrane protein composition and stability, and metabolism. Surprisingly, we show that absence of GLUT1 is well tolerated by differentiating erythroblasts, with no impairment of proliferation, enucleation, or tangible alterations to resulting reticulocyte membrane composition or deformability, challenging prevailing dogma surrounding the necessity of this abundant protein for RBC development, structure, and function.

## Materials and methods

### Source material

All human blood source material was provided with written informed consent for research use given in accordance with the Declaration of Helsinki (National Health Service Blood and Transplant [NHSBT], Filton, Bristol). The research into the mechanisms of erythropoiesis was reviewed and approved by the Bristol University research ethics committee (no. 12/SW/0199).

### Antibodies

See supplemental Methods for a detailed list of antibodies and reagents used.

### CD34^+^ and BEL-A culture

CD34^+^ cells were isolated, expanded, and differentiated as previously described^17^ and further detailed in supplemental Methods.

BEL-A (Bristol erythroid line–adult) cells were cultured as previously described.^18^^,^^19^ For expansion, cells were seeded at 0.5 × 10^5^ to 1 × 10^5^ cells per mL in expansion medium, consisting of StemSpan serum-free expansion medium (Stem Cell Technologies) supplemented with 50 ng/mL stem cell factor (SCF; R&D Systems), 3 U/mL erythropoietin (EPO, Neocormon), 1 μM dexamethasone (Sigma-Aldrich), and 1 μg/mL doxycycline (Clontech). Cells were incubated at 37°C, 5% CO_2_, with complete medium changes every 48 to 72 hours. BEL-A differentiation protocol was performed as described by King et al.^20^ Briefly, cells were seeded at 1.5 × 10^5^ cells per mL in primary differentiation medium supplemented with 1 ng/mL interleukin-3 (R&D Systems), 10 ng/mL SCF, and 1 μg/mL doxycycline. After 2 days, cells were reseeded at 3 × 10^5^ cells per mL in fresh medium. On day 4, cells were reseeded at 5 × 10^5^ cells per mL in fresh medium without doxycycline. On day 7, cells were transferred to tertiary medium (no SCF, interleukin-3, or doxycycline) at 1 × 10^6^ cells per mL. Another complete media change was performed on day 9. Cells were analyzed on day 10 or 11.

### CRISPR editing of cells

BEL-A cells were nucleofected using a 4-dimensional nucleofector system with 20 μL of Nucleocuvette Strip (Lonza) in combination with the P3 primary cell buffer kit (Lonza). Per sample, 2 × 10^5^ cells were transfected with preincubated 18 pmol of CRISPR-associated protein 9 (Cas9; TrueCut Cas9 Protein version, Thermo Fisher) and 45 pmol of either SLC2A1 targeting single-guide RNA (sgRNA; 5′-GGATGCTCTCCCCATAGCGG, Synthego) or nontargeting (NT) control (Negative Control Scrambled sgRNA#1, Synthego), using program DZ-100. For CD34^+^ isolated cells, nucleofection was performed on day 3 after isolation, in which 5 × 10^5^ cells were nucleofected with 50 pmol of Cas9 and 125 pmol of sgRNA using electroporation program EO-100.

### Flow cytometry and fluorescence-activated cell sorting

For flow cytometry, cells were fixed (1% paraformaldehyde, 0.0075% glutaraldehyde) to prevent antibody agglutination, except for assays using GLUT1 receptor binding domain (RBD; GLUT1.RBD). Samples were labeled with primary antibodies in PBSAG comprising phosphate-buffered saline (PBS) with 1% weight per volume (w/v) glucose and 0.5% (w/v) bovine serum albumin (BSA) supplemented with extra 1% (w/v) BSA for 30 minutes, in the dark. METAFORA’s GLUT1.RBD was incubated at 37°C, and all remaining antibodies incubated at 4°C. Cells were washed twice with PBSAG and, if required, incubated with appropriate secondary antibody under the same conditions as described for primary antibody. Cells were washed twice with PBSAG and analyzed on a Miltenyi MACSQuant 10 flow cytometer. Data were analyzed using FlowJo version 10.7 (FlowJo). Reticulocytes were identified by gating on the Hoechst-negative population.

Cells were sorted using a BD Influx Cell Sorter (BD Biosciences). BEL-A CRISPR–edited populations were single-cell sorted based on viability (DRAQ7 negativity). Primary GLUT1 KO cultures were sorted on days 6 or 7 of differentiation using DRAQ7 and the enhanced green fluorescent protein (eGFP) fused GLUT1.RBD to purify the negative population with a gate based on NT guide control cells.

### Osmotic fragility assays

Filtered reticulocytes (1 × 10^5^ to 2 × 10^5^ cells per well) were incubated in decreasing NaCl concentrations (0.9%-0%) for 10 minutes at 37°C. Lysis was stopped by adding 4× volume of PBSAG. Live cells, considered as having a normal forward scatter/side scatter profile as defined by the 0.9% NaCl control, were counted by flow cytometry using the MACSQuant10.^21^

### Automated rheoscopy

A total of 1 × 10^6^ cells were diluted in 200 μL of a polyvinylpyrrolidone solution (viscosity, 28.1 mPa/s; Mechatronics Instruments). Cell deformability distributions were assessed in an automated rheoscope and cell analyzer according to previously published protocols.^22^ At least 2000 valid cells per sample were analyzed.

### Lipid peroxidation

The assay was performed on prefiltered CD34^+^ cell–derived reticulocytes, in culturing media, using Hoechst stain to identify reticulocytes. C11-Bodipy (10 μM from a dimethyl sulfoxide stock, Invitrogen) was used as a fluorescent lipid peroxidation reporter molecule. Oxidation was induced by cumene-hydroperoxide (25 μM from an ethanol stock, Sigma). Ethanol was used as a vehicle control. The 3 reagents were added simultaneously and incubated for 30 minutes at 37°C. Samples were washed 3× with PBSAG and lipid peroxidation was measured on an MACSQuant10 (488 nm).

### Proteomics, metabolomics, and lipidomics

Samples were extracted and treated as extensively reported in supplemental Methods, which also includes details of the database searches and protein identification after proteomics mass spectrometry. Proteomics^23^ and metabolomics analyses were performed as previously described.^24^ Total lipids were extracted as previously described.^25^

## Results

**Figure 1.**
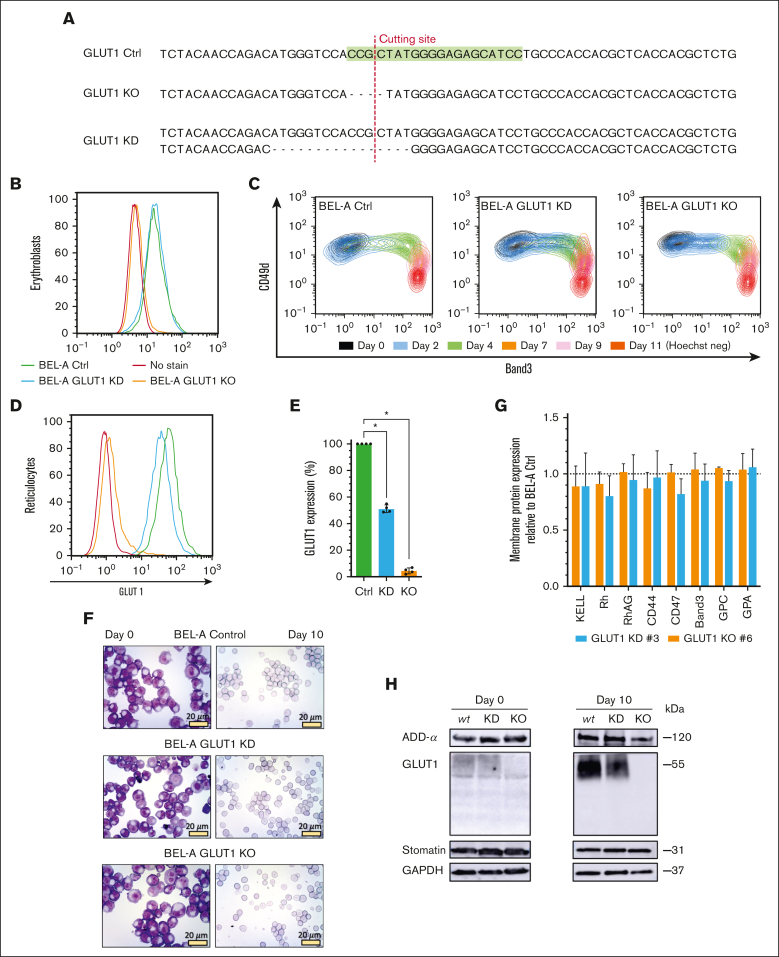
**CRISPR–mediated GLUT1 KO on immortalized BEL-A erythroblasts successfully generate reticulocytes.** (A) Sequencing of SLC2A1 (GLUT1) gene on Ctrl BEL-A, highlighting the guide RNA (green) used for CRISPR editing. Sanger sequencing of clonal edited lines shows a homozygous 4–base pair (bp) frameshift mutation on the GLUT1 KO line, and a heterozygotic 16-bp deletion on the KD line, both in the vicinity of the cutting site (red line). Flow cytometry histogram of GLUT1 staining in BEL-A erythroblasts (B) and derived reticulocytes (D) from Ctrl (green), GLUT1 KD (blue), and GLUT1 KO (orange) cell lines compared with no-stain control (red). Cells were stained with anti-GLUT1 eGFP conjugate. (C) Flow cytometry analysis of cell surface marker expression during differentiation. Cells were colabeled with anti-band3 primary antibody used in conjunction with an immunoglobulin G1 (IgG1) APC secondary and anti–α4-integrin FITC conjugate. For day 11, reticulocytes were identified using Hoechst as a nuclear DNA stain. (E) Bar graph illustrates the percentage GLUT1 expression on reticulocytes derived from indicated cell lines. Data are normalized to endogenous expression of Ctrl BEL-A from the median fluorescence intensity (n = 4). Individual data points are shown. Error bars indicate standard error of mean. (F) Representative images of May-Grünwald and Giemsa–stained cytospins depicting expanding BEL-A erythroblasts (day 0) and corresponding filtered reticulocytes after 10 day differentiation protocol; 40× original magnification. Scale bars, 20 μm, shown for each image. (G) Bar graphs illustrate expression of various membrane proteins on reticulocytes derived from indicated cell lines (n = 3). Reticulocytes were identified based on Hoechst stain negativity. Data are normalized to endogenous expression of Ctrl BEL-A and represents the median fluorescence intensity (n = 3). Individual data points are shown. Error bars indicate standard error of mean. (H) Western blots of lysates obtained from indicated cell lines at day 0 of differentiation and reticulocytes filtered after 10-day protocol, incubated with antibodies to α-adducin, GLUT1, stomatin, and glyceraldehyde-3-phosphate dehydrogenase (GAPDH; loading control). Multiple Mann-Whitney *U* tests were used to test for differences between groups. ∗*P* < .05. Error bars indicate standard deviation.

To explore the impact of complete loss of GLUT1 on human erythroid cells, CRISPR–mediated gene editing of the BEL-A cell line was first exploited to generate GLUT1 knockdown (KD) and KO erythroblast cell lines; disruptive monoallelic or biallelic mutations, respectively, were identified in clonal lines (Figure 1A; supplemental Figure 1). GLUT1 expression on expanding cells was measured by flow cytometry using a GLUT1–specific viral RBD.^26^^,^^27^ Figure 1B confirms a complete loss of expression in BEL-A KO cells, whereas the KD had no overall reduction of GLUT1 when compared with unedited BEL-A (control [Ctrl]). Real-time quantitative polymerase chain reaction (supplemental Figure 2) revealed a 70% increase in GLUT1 messenger RNA (mRNA) on the KD cells, and a 90% decrease for the KO cells. RNA levels of an alternative glucose transporter, GLUT3, previously reported to be expressed in erythroblasts were significantly upregulated in GLUT1-KO (eightfold upregulation) and KD (2.6-fold increase).

Because GLUT1 expression increases drastically during erythroid differentiation,^28^ the ability of GLUT1-KO erythroblasts to undergo terminal erythroid differentiation and enucleation was assessed through monitoring of band 3 and α4-integrin (CD49d).^29^ Figure 1C and supplemental Figure 3 illustrate the ability of both GLUT1-KD and GLUT1-KO cells lines to differentiate producing the same cascading pattern as the BEL-A Ctrl cells. GLUT1 expression was assessed on both differentiating erythroblasts and enucleated reticulocytes (Figure 1D-E). Although there was no observed reduction in expression at the erythroblast stage, the heterozygous GLUT1 mutation produced reticulocytes with a 49.2% decrease in surface GLUT1. May-Grünwald and Giemsa–stained cytospins of expanding erythroblasts and reticulocytes (Figure 1F) did not identify discernible cellular morphological differences arising from GLUT1 deficiency or absence. Figure 1G illustrates no reductions in surface expression of prominent membrane proteins compared with that of Ctrl cells. Complete absence or reduction of GLUT1 was confirmed by immunoblotting and further validated through proteomics (supplemental Figure 4). Reported GLUT1 interacting proteins adducin-α and atomatin were unaltered in expression (Figure 1H). GLUT4 was detected through proteomics despite its low abundance, with no increased expression in either GLUT1-KD or GLUT1-KO cells. Conversely, GLUT3 was absent in the proteomic data.

**Figure 2.**
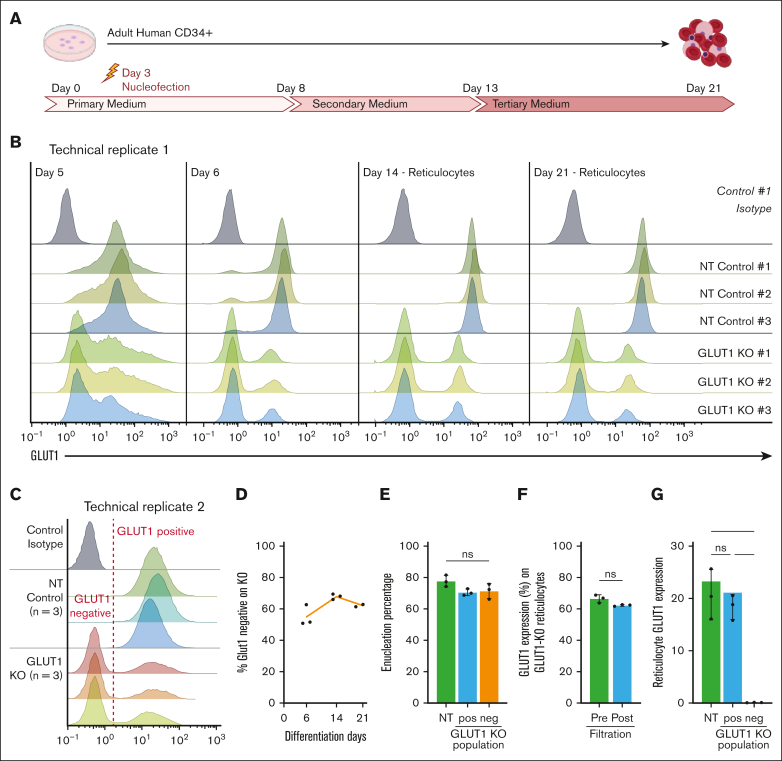
**Primary erythropoiesis is not affected by GLUT1 KO.** (A) Schematic diagram of human CD34^+^ 3-step culture method. PBMCs are isolated from apheresis cones, followed by CD34^+^ magnetic separation. Cells are then nucleofected on day 3 with NT or GLUT1–specific sgRNAs. (B) Flow cytometry histograms show GLUT1 expression of 3 donors and nucleofected with NT or SLC2A1 targeting sgRNAs on days 5, 6, 14, and 21 of differentiation. For days 14 and 21, Hoechst stain was used to identify the reticulocytes. Cells were stained with anti–GLUT1 FITC conjugate (n = 3) or a no-stain control (black). (C) Day-21 filtered reticulocytes stained with anti–GLUT1 FITC conjugate (n = 3) or a no-stain control (black). (D) Percentage of GLUT1–negative population on GLUT1–targeted KO (n = 3) on days 6, 14, and 21 of differentiation. (E) Percentage of reticulocytes (Hoechst stain negative) at day 21 of differentiation on NT control and negative and positive GLUT1 populations of the GLUT1–targeted KO. (F) Percentage of GLUT1–negative population on GLUT1–targeted KO on reticulocytes before and after filtration. (G) Bar graph illustrates GLUT1 expression on reticulocytes from NT and GLUT1–negative and –positive populations of the GLUT1–targeted KO. Data represent the median fluorescence intensity (n = 3). Individual data points are shown. Multiple Mann-Whitney *U* tests were used to test for differences between groups. ∗*P* < .05. Error bars indicate standard deviation.

To determine whether compensation for GLUT1 absence is enabled by prolonged expansion of the BEL-A cell line, CRISPR KO was performed on cultured primary hematopoietic progenitors nucleofected 3 days after isolation, as indicated (Figure 2A). Cells were cultured for 21 days and GLUT1 expression assessed by flow cytometry throughout differentiation (Figure 2B). Note that 48 hours after nucleofection (day 5) it is already possible to discriminate between NT controls and GLUT1–targeted populations with a clear negative comprising ∼75% of the total cells discernible on day 6.

GLUT1 expression was measured on Hoechst stain–negative reticulocytes present at days 14 and 21 with the same proportion (75%) of cells shown to be GLUT1 negative as on day 6. Of note, assessment of the GLUT1–positive population within the CRISPR–edited samples reveals a decreased GLUT1 expression when compared with the matched NT control, signifying a KD population. It is noteworthy that cells from all 3 donors enucleated and from day 6 of erythroid differentiation maintained a stable GLUT1–negative population, indicating no competitive disadvantage in erythroblast expansion resulting from absence of GLUT1.

We performed a replicate assay of the CRISPR KO (Figure 2C-G), resulting in an average of 62% GLUT1–negative population across 3 donors. Here, within the GLUT1–positive population, expression of GLUT1 is comparable with that on the matched NT controls. By colabeling day-21 prefiltration samples with anti-GLUT1.RBD and Hoechst it was possible to compare enucleation of GLUT1–positive and –negative populations within GLUT1–targeted cultures, as well as with the NT controls (Figure 2E). There is no significant difference between the 3 populations, demonstrating that the absence of GLUT1 does not adversely affect the membrane remodeling and cytoskeletal changes required for nucleus extrusion. To assess the effect of GLUT1 absence on physical membrane properties of reticulocytes, day-21 cultures were filtered using a 5-μm filter with the GLUT1–negative cells of each KO-targeted donor assessed before and after filtration to establish differential ability to pass through the filter (Figure 2F). No significant difference in proportion of GLUT1–negative cells before and after filtration was observed (66.5% ± 2.6% vs 62.3% ± 0.6%), indicating that absence of GLUT1 does not affect reticulocyte capacity to traverse 5-μm pores.

**Figure 3.**
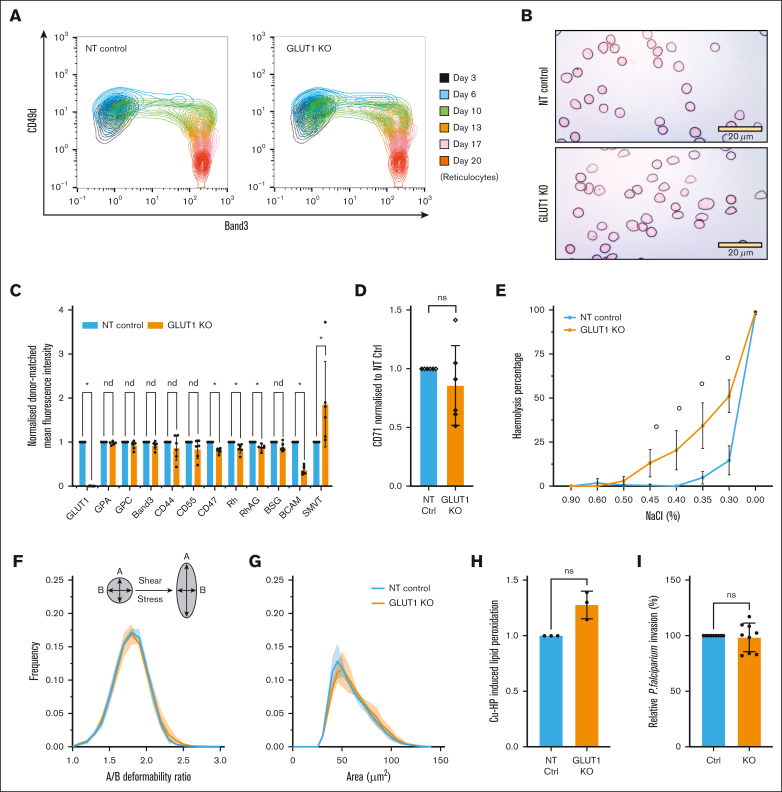
**Properties of CD34**^**+**^**GLUT1-KO–derived reticulocytes.** (A) Waterfall plot indicating progression of control and GLUT1-KO–transfected cultures of single donor CD34^+^ differentiation. Cells were colabeled with anti-band3 primary antibody used in conjunction with an IgG1 APC secondary and anti–α4-integrin FITC conjugate. For day 20, reticulocytes were identified using Hoechst stain as a nuclear DNA stain. (B) Representative cytospins of the same culture were obtained on day 20 after leukofiltration. 40× original magnification. Scale bars, 20 μm, shown for each image. (C) Bar graphs indicate the expression of various membrane proteins on reticulocytes derived from NT or GLUT1-KO primary cultures (n = 6, for SMVT n = 3, with 2 technical repeats each). Significance was assessed by multiple Mann-Whitney *U* tests with a false discovery rate of 1% to account for multiple comparisons. “∗” shows q < .01 and “nd” shows q > .01. (D) Anti-transferrin receptor (CD71) labeling of on reticulocytes (n = 6, open or filled dots indicate 2 separate cultures, each with 3 donors). For both, reticulocytes were leukofiltered on day 20. Data are normalized to each donor-matched NT control and represents the median fluorescence intensity. (E) Osmotic resistance analysis calculated based on viable cell counts (flow cytometer) after incubation with decreasing concentrations of NaCl (n = 3, 2 technical replicates, “∘” *P* ≤ .0021). (F) Deformability and (G) cell area (μm^2^) were measured under shear stress by automated rheoscope cell analyzer (n = 3, N > 2000 cells), which elongates cells and measures length over width as deformability parameter. Shaded region represents the standard deviation. (H) Quantitative analysis of lipid peroxidation detected by a shift in the fluorescence signal after treatment with 25 mM cumene hydroperoxide. Data normalized to each donor-matched NT control (n = 3, 3 technical replicates). (I) Bar graph quantifying *P falciparum* reticulocyte invasion efficiency. Invasion was assessed by flow cytometry using a SYBR-green DNA stain (3 separate parasitemia percentages, 3 technical replicates) and data were normalized per matched-donor (n = 3) NT control. This figure comprises data obtained from 2 independent cultures, each of 3 donors with panels A-B presenting representative data from cultures with fluorescence-activated cell sorted GLUT1-KO purity of 99%; panels E-I 94% and panels C-D combined data from all 6 donors. A nonparametric Mann-Whitney *U* test or Kruskal-Wallis test with Bonferroni correction were used to test for differences between groups when not specifically mentioned, ∗*P* < .05, ∗∗*P* < .01, and ∗∗∗*P* < .001. Error bars indicate standard deviation.

To further investigate the properties of GLUT1–negative reticulocytes, additional KOs were performed and enriched for using fluorescence-activated cell sorting. As previously illustrated for the BEL-A GLUT1 KO, no substantial differences were identified in terminal erythroid differentiation per CD49d/band 3 labeling (Figure 3A; supplemental Figure 5), nor did cell morphology change between CD34^+^ NT and GLUT1-KO populations (Figure 3B). The mRNA levels from day-7 cells were analyzed by real-time quantitative polymerase chain reaction (supplemental Figure 6) revealing a 61.3% ± 8.8% (standard deviation) decrease in GLUT1 transcripts in the GLUT1-KO population when compared with matched NT controls. Of note is the significant 1.57 ± 0.38 (standard deviation)–fold increase in GLUT3 mRNA in the KO group. Expression of surface membrane proteins was analyzed by flow cytometry, with the median fluorescence intensity averaged for the NT controls (Figure 3C). Complete absence of GLUT1 surface expression was confirmed, and no alterations in expression of most major erythrocyte membrane proteins as indicated was observed. Within the NT controls, the expression of BCAM (basal cell adhesion molecule, or Lutheran blood group protein^30^) exhibits considerable variability because of a well-established donor-variable dual population of Lu-presenting cells (supplemental Figure 7). However, even considering this variability, there remains a statistically significant reduction in BCAM expression in the GLUT1-KO compared with the matched NT cultures. Also, significantly reduced, albeit mildly, are CD47, Rh, and RhAG (components of the Rh subcomplex).

Expression of various cell surface nutrient transporters was also measured, using RBDs that function as specific ligands of solute carrier (SLC) nutrient transporters.^31^ Among these, the sodium-dependent multivitamin transporter (SMVT; product of the SLC5A6 gene) is the only protein significantly increased in the GLUT1-KO samples using available reagents. Data on the remaining nutrient transporters can be seen in supplemental Figure 8. Reticulocyte maturity as assessed by CD71^32^ (transferrin receptor) levels varied between cultures but was not consistently or significantly altered by the absence of GLUT1 (Figure 3D).

**Figure 4.**
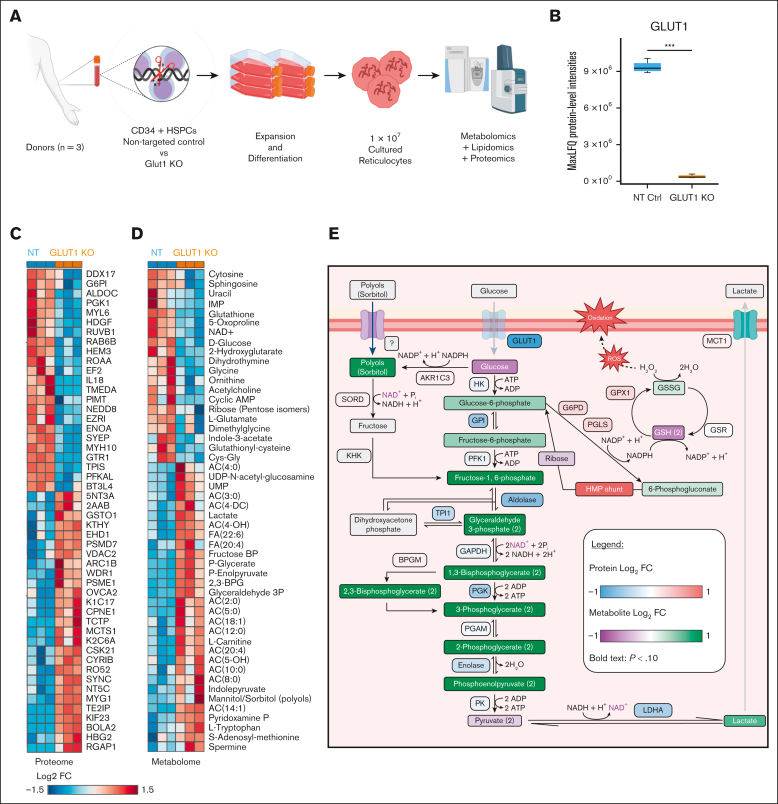
**Proteomics confirms GLUT1 absence in CD34**^**+**^**GLUT1-KO–derived reticulocytes and metabolite analyses reveals downregulated metabolic processes.** (A) Simplified schematic of multiomics cell preparation, in which CD34^+^ from 3 donors were nucleofected with either GLUT1–targeting or NT sgRNAs, expanded and differentiated into reticulocytes; 10 million filtered reticulocytes were needed for comprehensive analyses of the proteome, metabolome, and lipidome. (B) Box plot comparing GLUT1 protein level between NT and GLUT1-KO reticulocytes as maximum label-free quantification (MaxLFQ) protein-level intensities. Box plot analysis (mean ± minimum to maximum with standard deviation) was performed by RStudio, and significance was calculated upon false discovery rate correction (∗∗∗*P* < .001). (C-D) Hierarchical clustering of the top 50 *t* test significant proteins (C) and metabolites (D) between NT and GLUT1-KO CD34^+^-derived reticulocytes. (E) Schematic representation of glycolysis, the polyol pathway, and the glutathione redox cycle in which proteins and metabolites are color-coded by log_2_ (fold change) of GLUT1-KO reticulocytes in relation to NT control. The 10 steps of glycolysis are represented, with glucose and lactate both reduced in the KO whereas the remaining intermediate products increased. All involved enzymes are decreased (HK, hexokinase; GPI, glucose-6-phosphate isomerase; PFK1, phosphofructokinase-1; TPI1, triosephosphate isomerase; BPGM, biphosphoglycerate mutase; PGK, phosphoglycerate kinase; PGAM, phosphoglycerate mutase; PK, pyruvate kinase; and LDHA, lactate dehydrogenase A). There is an imbalance in the glutathione cycle, as a consequence of increased reactive oxygen species (ROS), characterized by the depletion of reduced glutathione (GSH) and increase of oxidized glutathione (GSSG) and glutathione peroxidase 1 (GPX1). The hexose monophosphate (HMP) shunt is upregulated as a source of reduced NAD phosphate (NADPH), needed to maintain glutathione in its reduced form. An increase in polyols (such as sorbitol and mannitol) was also detected, which can be converted into fructose-1,6-phosphate by sorbitol dehydrogenase (SORD) and ketohexokinase (KHK). G6PD, glucose-6-phosphate dehydrogenase; PGLS, 6-phosphogluconolactonase; GSR, glutathione-disulfide reductase. Created with BioRender.

To provide a global overview of alterations to protein expression, matched NT-control and GLUT1-KO reticulocytes (n = 3) were subjected to proteomic analysis (Figure 4). Deficiency of GLUT1 was confirmed in GLUT1 KO, with a 26-fold change decrease in the GLUT1-KO reticulocytes compared to NT-controls (Figure 4B; *P* < .001). GLUT3 and GLUT4 were detected despite their low abundance but showed no increased expression in the KOs (supplemental Figure 9). No specific vitamin C or DHA transporters were identified, nor was SMVT detected in the data set. Proteins known to interact with GLUT1, α- and β-adducin, dematin, and stomatin, show no significant alteration in expression in the KO samples. CD47, Rh (RhD and RhCE), RhAG, and BCAM also show no significant changes in total protein content despite the reduction observed in surface levels. However, gene ontology enrichment analysis revealed a highly significant enrichment of glycolysis-associated proteins (*P* < 10^10^), with downregulation of the entire glycolytic pathway, and conversely an upregulation of AMP-activated protein kinase signaling– and autophagy-associated proteins, promoting catabolic pathways to generate more ATP.

In vivo, RBCs remain in circulation for up to 120 days, being continuously exposed to shear stress and repeated elastic deformations. Given the varied potential contributions to RBC stability that arise from GLUT1 influence on membrane-cytoskeletal connectivity, solute transport, and metabolism, we used a variety of in vitro techniques designed to dissect the effects of absence of GLUT1 in reticulocytes. Osmotic fragility assays summarized in Figure 3E illustrate increased hemolysis compared with control cells, evident at 0.45% NaCl and maintained at lower NaCl levels, indicative of a higher osmotic fragility on GLUT1-KO cells and suggestive of a potential defect in volume homeostasis or ion balance.

GLUT1, via association with adducin and dematin, is reported to provide a site of vertical attachment between membrane and cytoskeleton in RBCs.^5^ To investigate cell deformability and membrane stability, cells were subjected to automated rheoscope and cell analyzer.^22^ Figure 3F-G indicate no differences in cross sectional area or capacity to undergo deformation (elongation index) arising from the absence of GLUT1, illustrating no detectable reductions in deformability.

In addition to its role in glucose transport, GLUT1 also acts as a transporter of DHA, which gets converted into ascorbic acid in the cytoplasm, a potent antioxidant.^33^ Using C11-Bodipy as a lipid peroxidation sensor we measured reactive oxygen species response using cumene hydroperoxide as an oxidizing agent. Although Figure 3H shows a small increase in lipid peroxidation (*P* = .0726) in the KO population, the range of individual data points precludes it from reaching statistical significance. As an additional assay to exploit the generation of this novel GLUT1 KO cell resource, reticulocytes were subjected to invasion assays with the malaria parasite *Plasmodium falciparum*. Interestingly, despite the abundance of GLUT1 within the reticulocyte/erythrocyte membrane, absence of GLUT1 did not impair the ability of reticulocytes to support invasion by *P falciparum*, with no significant differences in invasion efficiency of GLUT1 KO compared with NT control reticulocytes (Figure 3I), indicating that GLUT1 is not exploited as an essential receptor for merozoite invasion.

**Figure 5.**
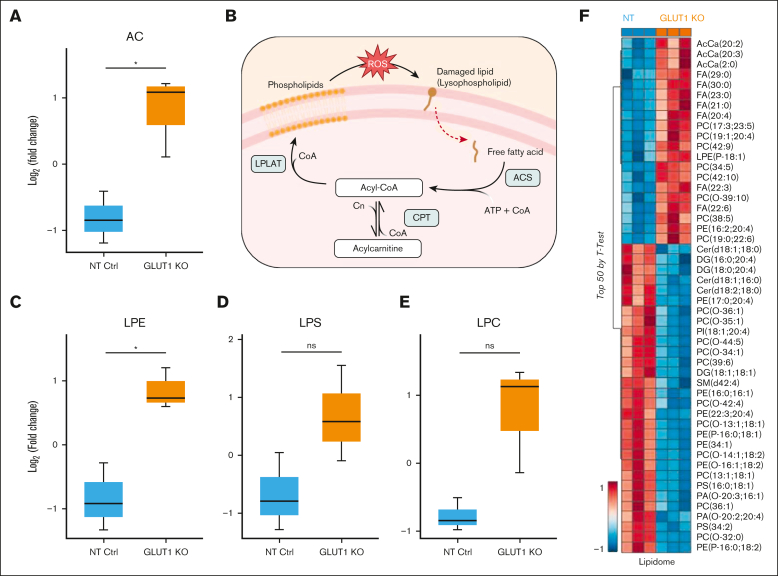
**Lipid composition highlights increased oxidant stress to the membrane of GLUT1-KO reticulocytes.** (A) Box plot showing the comparison of acyl-carnitines as log_2_ (fold change, FC), between NT and GLUT1-KO reticulocytes. Box plots analysis (mean ± minimum to maximum with standard deviation) was performed by RStudio, and significance was calculated upon false discovery rate correction (*P* < .05). (B) Simplified schematics of the Lands cycle, capable of repairing damaged lipids (lysophospholipids) generated from increased oxidative stress. The damaged chain is removed by a phospholipase, originating a free fatty acid, which is converted into acyl-CoAs by acyl-CoA synthetase (ACS) in an ATP-dependent reaction. Acyl-CoAs can be converted into acyl-carnitines by carnitine palmitoyltransferase (CPT). Lysophospholipid acyltransferases (LPLATs) incorporate undamaged fatty acid chains into membrane lysophospholipids, repairing the lipid membrane. (C-E) Box plots comparing lysophosphatidylethanolamine (LPE, C), lysophosphatidylserines (LPS, D), and lysophosphatidylcholine (LPC, E) between NT and GLUT1-KO reticulocytes, presented as log_2_(FC). (F) Hierarchical clustering of the top 50 *t* test significant lipids between NT and GLUT1-KO CD34^+^-derived reticulocytes. Created in BioRender.

The apparent absence of phenotype arising from the loss of such an upregulated and abundantly expressed protein within the erythroid lineage was surprising. To further explore potential distinctions that arise from the absence of GLUT1, the samples already tested for proteomic differences were also investigated to uncover metabolomic and lipidomic disparities (Figures 4 and 5). Interestingly, metabolomics analyses revealed that GLUT1-KO reticulocytes do compensate for a decrease in glucose uptake, as inferred by a significantly lower steady state levels of glucose and by upregulating glucose catabolism downstream of hexose phosphate. Figure 4D summarizes the metabolomic and proteomic alterations observed in the GLUT1-KO cells. Indeed, in GLUT1-KO cells, we detected significant elevation in the levels of fructose bisphosphate, glyceraldehyde 3-phosphate, 2,3-bisphosphoglycerate, phosphoglycerate (2 and 3 isomers), and phosphoenolpyruvate. However, despite net significant increases in lactate, decreases in pyruvate were observed, suggestive of altered pyruvate to lactate ratios, perhaps because of decreased nicotinamide adenine dinucleotide (NAD) + hydrogen (NADH) dependent methemoglobin reductase activity, which could outcompete lactate dehydrogenase for the same cofactor. Despite higher levels of virtually all glycolytic intermediates, especially at the payoff steps of glycolysis, no significant changes were observed between the 2 groups, suggesting that, in the absence of perturbation, increased glycolytic fluxes may compensate for decrease glucose uptake in the KO reticulocytes. Interestingly, elevation in polyols such as sorbitol, which can enter glycolysis at the fructose bisphosphate level, are suggestive of alternative sugar substrates that could contribute to compensating for the partially ablated glucose uptake.

Despite these adaptations, markers of oxidant stress were observed in the GLUT1-KO group, even in the absence of perturbation. For example, KO cells showed significantly lower levels of reduced glutathione, with near significant (*P* = .06) elevation in oxidized to reduced glutathione ratios (supplemental Figure 10), suggestive of a rewiring of glucose consumption mainly through glycolysis, perhaps via the reduction of glucose oxidation via the pentose phosphate, a key pathway for the generation of the reducing equivalent reduced NAD phosphate, which is directly or indirectly involved in virtually all antioxidant reactions in the mature erythrocyte.

Finally, and most evidently, L-carnitine and all acyl-carnitines were found to increase in the KO reticulocytes (Figure 5A). In mature RBCs this class of metabolites is associated with lipid membrane damage repair via the Lands cycle,^34^^,^^35^ represented in Figure 5B. Lipidomics results indicated significant decreases in the levels of cholesteryl-esters, diacylglycerols, ceramides, and all phospholipids (phosphatidylcholine [PC], phosphatidylethanolamine [PE], phosphatidylinositol [PI], phosphatidylserine [PS], phosphatidylglycerol [PG], and phosphatidic acid [PA]) in the KO group, except for triacylglycerols, free fatty acids, and monohexosylceramides (supplemental Figure 11). Most importantly, elevation in all lysophospholipids (significant for lysophosphatidylethanolamine, borderline significant for lysophosphatidylserines and lysophosphatidylcholine, as seen in Figure 5C-E) are suggestive of increased phospholipase activity upon activation of the Lands cycle for the repair of damaged lipids in the KO group.

## Discussion

As 1 of the most abundantly expressed proteins within the erythrocyte membrane, GLUT1 is widely assumed to play a crucial role in the development, structure, and function of the RBC. However, suitable erythroid models to study the effect of lack of GLUT1 in humans is lacking. Mouse models are inappropriate owing to the absence of GLUT1 in the adult mouse erythrocyte^2^ and, to date, in human erythroid cells only a 60% KD has been achieved,^36^ levels that mimic the naturally occurring G1DS,^27^ a disease that is hematological symptomless despite presentation of neurological phenotypes. Here, we demonstrate the ability to generate completely GLUT1–deficient erythroid cells that successfully complete terminal differentiation to generate enucleated reticulocytes unexpectedly deficient in phenotype, rewriting our understanding of the essentiality of this protein in human erythroid biology. RBC structural integrity and capacity for deformation arise from vertical protein associations between integral membrane proteins and the underlying spectrin-based cytoskeleton, mediated via an array of cytoskeletal adapter proteins. It would not be unreasonable to hypothesize that loss of GLUT1, a protein that accounts for ∼10% of the protein component of the RBC membrane,^37^ would confer disruption to membrane integrity. Surprisingly, however, there were no reductions in reported GLUT1–associating proteins, nor any other detectable membrane or cytoskeletal protein were observed (aside from a mild reduction in Rh subcomplex components). Furthermore, neither deformability, as assessed through rheoscopy, nor ability to support invasion by *P falciparum* was found to be altered in GLUT1-KO reticulocytes. No significant compensatory upregulation of membrane proteins that could be anticipated to occupy vacant binding sites or residency within the plasma membrane was observed through proteomics, indicating that GLUT1 does not play a direct role in maintenance of the structural integrity of the RBC via its protein–protein interactions.

Metabolomic studies revealed that GLUT1-KO reticulocytes exhibit hallmarks indicative of reduced glucose import with downregulated metabolic processes and upregulated adenosine monophosphate (AMP)-activated protein kinase signaling, consistent with reduced glucose content. Surprisingly, the absence of GLUT1 does not present an obvious impediment to erythroblast terminal differentiation or enucleation; this may reflect initial compensation of glucose import mediated via GLUT3, which has been previously demonstrated to be expressed in the early stages of differentiation before loss and replacement by GLUT1,^28^ and is corroborated by the *SLC2A3* transcriptional upregulation detected in the expanding erythroblasts of GLUT1-KO BEL-A and primary cells. Although increased activity of alternative glucose transporters expressed at residual levels cannot be excluded, no upregulation of GLUT3 or GLUT4 in differentiated reticulocytes was detected by proteomics, nor increased abundance of mitochondria to maximize energy output, as inferred from comparable levels of mitochondrial proteins via proteomics between NT and GLUT1-KO cells. Thus, our data show, to our knowledge, for the first time, that GLUT1 is not required for erythroid cells to obtain sufficient glucose to drive terminal differentiation.

Although GLUT1 is among the proteins most upregulated during erythropoiesis, seminal work from Oburoglu et al^36^ reported a lack of erythropoiesis defects when blocking glucose catabolism in early progenitors, and Montel-Hagen et al^10^ demonstrated that glucose transport actually decreases during the latter stages of erythroid differentiation, replaced instead by transport of DHA, an oxidized form of ascorbic acid in a switch regulated by stomatin association. Absence of GLUT1 is therefore predicted to also affect intracellular antioxidant levels and redox balance. Interestingly, contrastingly to the unaltered deformability, increased osmotic fragility of GLUT1-KO reticulocytes compared with unedited controls was detected, mimicking a phenotype observed in RBCs of mice with reduced ascorbic acid intake.^38^^,^^39^ Metabolomics also revealed a depletion of the glutathione pool. These changes were accompanied by the maintenance of total ATP levels with increased steady state levels of glycolysis intermediates downstream to fructose bisphosphate, elevation in the levels of alternative sugar substrates like sorbitol, and decrease in the levels of pentose phosphate pathway metabolites.

Absence of GLUT1–mediated DHA transport likely adds pressure to the remaining cellular antioxidant defense mechanisms while trying to maintain redox balance, as suggested by the borderline (*P* = .07) decreases in DHA in the KO cells. Of note, in a background of minimal detectable compensatory alterations, we did observe increased expression of the SMVT, which catalyzes the uptake of α-lipoic acid (a potent antioxidant), pantothenic acid (vitamin B5, precursor to coenzyme A that participates in heme synthesis^40^ and the Lands cycle), and biotin^41^ in primary GLUT1-KO reticulocytes. Our data indicate a mild and potentially partially compensated defect in antioxidant capacity of GLUT1-KO reticulocytes.

Altogether, these results indicate that, although nonlethal in the absence of perturbations, the GLUT1-KO mutation introduces a strain to RBC antioxidant metabolism by forcing glycolytic metabolism through the Embden-Meyerhof Parnas pathway at the expense of antioxidant pathways such as the hexose monophosphate shunt. Interestingly, these metabolic alterations were accompanied by elevation in acyl-carnitine pools, lysophospholipids, and odd-chain fatty acids derived from α-oxidation of longer-chain polyunsaturated fatty acids, despite decreases in several classes of lipids. These lipidomics data are suggestive of increased oxidant stress to the RBC membrane fraction, consistent with the activation of the Lands cycle and an increased susceptibility to osmotic fragility, similar to that observed for RBCs from patients suffering from chronic^42^ or acute kidney disease,^43^^,^^44^ sickle cell disease, and hypoxia.^45^

In vivo circulating RBCs are highly exposed to oxidative stress with repetitive nature of erythrocyte function hard to replicate in vitro, such as the accumulation of reactive oxygen species and consequent repetitive deformability decreases. Although experimentally impractical to explore, we recognize that as reticulocytes derived through in vitro culture, the full effects of complete GLUT1 deficiency subject to the rigors of in vivo circulation and in the absence of residual mitochondria (loss of which is induced by circulation) may not be evident. We predict that GLUT1 KO will most likely exhibit a reduced in vivo circulatory half-life as a consequence of increased osmotic fragility, altered energy metabolism, and disrupted redox balance observed here.

These data substantially extend our understanding of the role glucose transport and metabolism play in erythropoiesis and redefines the importance of GLUT1 in RBC membrane structure. Furthermore, for many years, the absence of hematological phenotype associated with reduced GLUT1 expression in G1DS has remained unexplained, attributed to the remaining copies of the transporter still present. In generating completely deficient GLUT1 erythroid cells that demonstrate no apparent defects in expansion, enucleation, or structural phenotypes, we now provide cell biological evidence that supports the clinical consensus that a reduction in GLUT1 abundance in RBCs does not cause anemia in G1DS.

Conflict-of-interest disclosure: A.M.T. is a cofounder of, a director for, and consultant to Scarlet Therapeutics Ltd. T.J.S. is a cofounder of and scientific consultant to Scarlet Therapeutics Ltd. The remaining authors declare no competing financial interests.
